# Clinical features, angio-architectural phenotypes, and treatment strategy of foramen magnum dural arteriovenous fistulas: a retrospective case series study

**DOI:** 10.3389/fneur.2023.1121075

**Published:** 2023-04-18

**Authors:** Zhipeng Xiao, Weizhen Gao, Hongyu Zhou, Xiaohua Zhang, Jiong Dai, Jieqing Wan, Liemei Guo

**Affiliations:** Department of Neurosurgery, Renji Hospital, Shanghai Jiaotong University School of Medicine, Pudong, Shanghai, China

**Keywords:** foramen magnum DAVF foramen magnum, dural arteriovenous fistula, subarachnoid hemorrhage, myelopathy, Hybrid Angio-Surgical Suite

## Abstract

**Background:**

The rarity and complex angioarchitecture of foramen magnum dural arteriovenous fistulas (DAVFs) make its treatment difficult and controversial. We aimed to describe their clinical features, angio-architectural phenotypes, and treatments, through a case series study.

**Methods:**

We first retrospectively studied cases of foramen magnum DAVFs treated in our Cerebrovascular Center, and then reviewed the published cases on Pubmed. The clinical characteristics, angioarchitecture, and treatments were analyzed.

**Results:**

A total of 55 patients were confirmed with foramen magnum DAVFs, which included 50 men and 5 women, with a mean age of 52.8 years. Most patients presented with subarachnoid hemorrhage (SAH) (21/55) or myelopathy (30/55), depending on the venous drainage pattern. In this group, 21 DAVFs were supplied by only the vertebral artery (VA), three by only the occipital artery (OA), three by only the ascending pharyngeal artery (APA), and the remaining 28 DAVFs were supplied by two or three of these feeding arteries. Most cases (30/55) were treated with only endovascular embolization, 18 cases (18/55) with only surgical disconnection, five cases (5/55) with combined therapy, and two cases rejected treatment. The angiographic outcome of complete obliteration was achieved in most patients (50/55). In addition, two cases of foramen magnum DAVFs were treated by us in a Hybrid Angio-Surgical Suite (HASS) with good outcomes.

**Conclusions:**

Foramen magnum DAVFs are rare and their angio-architectural features are complicated. The treatment option (microsurgical disconnection or endovascular embolization) should be weighed carefully, and combined therapy in HASS could be a more feasible and less invasive treatment option.

## Introduction

Foramen magnum dural arteriovenous fistula (DAVF) is a subset of craniocervical DAVF with the fistula point around the foramen magnum. Generally, the foramen magnum region lies in the bilateral occipital area that runs laterally up to the jugular foramen and includes both the inner and outer sides of the occipital bone (1). Most foramen magnum DAVFs are presented with myelopathy or subarachnoid hemorrhage, depending on the pattern of venous drainage (1). Foramen magnum DAVFs are rare, representing only 1.5–2.3% of all intracranial DAVFs (1, 2), and their rarity and complicated angioarchitectures make the treatment difficult and controversial.

Generally, microsurgical disconnection of the shunt was the predominant treatment option for the foramen magnum DAVFs before 2010 (3–5). However, in the past decade, with remarkable advances in the endovascular techniques providing highly flexible hydrophilic-coated catheters and new non-adhesive liquid embolic agents, such as Onyx and N-butyl-cyanoacrylate (NBCA) glue, endovascular embolization of the fistula has become an alternative option for treating the foramen magnum DAVFs (6–8). It has been reported that ~80% of the foramen magnum DAVFs in the literature were treated endovascularly (9). Nevertheless, the incomplete obliteration and reoccurrence of the fistula after endovascular embolization could not be underestimated, and some cases even required staged or salvage combined surgeries (1, 10). Recently, a combined surgical-endovascular technique in a hybrid operating room (Hybrid Angio-Surgical Suite, HASS) has emerged as a solution to the complexity of cerebrovascular surgery, and this technique has been an effective treatment option for DAVFs complicated by inaccessible arterial and transvenous approaches (11, 12). Coincidentally, we applied this concept of a HASS to treat two complicated cases of foramen magnum DAVFs with good outcomes.

In this retrospective observational case series, we first studied the cases of foramen magnum DAVFs treated in our Cerebrovascular Center, and then reviewed the published cases of foramen magnum DAVFs (4, 5, 7–10, 13–29), aiming to obtain a comprehensive understanding of the clinical symptoms, angioarchitectures, pathophysiological mechanisms and treatments for foramen magnum DAVFs.

## Methods

### Patients

In this retrospective case series, we reviewed clinical charts, radiological images, and operative notes of 13 consecutive patients who were diagnosed with foramen magnum DAVFs from January 2002 until April 2021. All patients were managed at the Neurosurgery Department, Renji Hospital, Shanghai Jiao Tong University School of Medicine. All available imaging studies were reviewed and analyzed. All the patients had digital subtraction angiography (DSA) of the bilateral internal and external carotid arteries and the vertebral arteries that carefully analyzed the arterial feeders, the location of the fistula, and the venous drainage patterns. Patients who presented with myelopathy had a complete spine MRI and spine DSA. All the patients gave consent to be enrolled and have their data published.

In addition, we found 78 published reports when we searched for “dural arteriovenous fistula foramen magnum” on PubMed, 26 of which included sufficient clinical descriptions and adequate angiographic architecture of the foramen magnum DAVFs.

### Data collection

The variables collected and analyzed include demographic profiles such as age and gender, clinical presentation and the duration of symptoms, and angio-architectural features of the fistulas including the location, arterial supply, venous drainage pattern, and presence of venous aneurysms. Treatment modalities, outcomes, and follow-up information were also documented and analyzed.

### Data analysis

All the data were analyzed and interpreted by the senior investigator with extensive knowledge of vascular neuroanatomy and angio-architectural interpretation. No statistical analysis software was warranted.

## Results

There were 55 patients (including 13 of our current cases) who were angiographically confirmed with foramen magnum DAVFs, which included 50 men (90.9%) and 5 women (9.1%). The mean age was 52.8 years, ranging from 20 to 83 years (Supplementary Table 1).

### Clinical presentation

Most patients presented with subarachnoid hemorrhage (SAH) or myelopathy (debility of bilateral limbs and/or urinary retention) after symptoms appeared, with 30 cases (54.5%) of myelopathy, 21 cases (38.2%) of SAH, and 4 cases (7.3%) of other etiologies (three intracranial hematomas, and one trigeminal neuralgia).

### Angioarchitecture

Generally, foramen magnum DAVFs have three important arterial supplies: the neuromeningeal trunk of the ascending pharyngeal artery (APA), the meningeal branches of the vertebral artery (VA), and the mastoid branches of the occipital artery (OA). As listed in Table 1, of the 55 cases in this case series study, 21 cases were supplied by only the VA; 3 cases were supplied by only the OA; 3 cases were supplied only by the APA, and the remaining 28 cases were supplied by two or three of these feeding arteries. Thus, more than half of these cases were not supplied by only one feeding artery, but by two or even three feeding arteries. In addition, as listed in Table 1, foramen magnum DAVFs usually first drain into the bridging medullary veins, but some DAVFs then drain into the intracranial cerebral veins or the spinal veins.

**Table 1:** Clinical and angioarchitectural characteristics and treatments of foramen magnum DAVFs.

	**Case**	**Age**	**Gender**	**Clinical presentation**	**Feeding arteries**	**Venous drainage**	**Treatment**	**Angiographic outcome**
Guo et al. (3)	1	47	M	SAH	VA	MV	Surgical disconnection	CO
	2	51	M	SAH	VA	MV	Surgical disconnection	CO
	3	35	M	SAH	OA, APA	MV, COS	Surgical disconnection	CO
	4	40	M	SAH	OA	MV, SS, COS	Treatment rejected	No follow up
Guo et al. (6)	5	45	M	SAH	VA	MV, COS	Embolization	CO
Present cases	6	30	M	SAH	VA	MV, COS	Treatment rejected	No follow up
	7	36	M	SAH	VA	MV	Surgical disconnection	CO
	8	40	M	SAH	VA	MV	Surgical disconnection	CO
	9	53	F	SAH	OA	COS	Embolization	CO
	10	52	M	SAH	VA	COS	Embolization	CO
	11	63	M	SAH	VA	MV	Surgical disconnection	CO
	12	62	M	Myelopathy	VA	MV, ASV	Surgical disconnection in HASS	CO
	13	60	M	SAH	VA	MV	Embolization in HASS	CO
Rivierez et al. (13)	14	50	M	SAH	VA	MV	Surgical disconnection	NR
Slaba et al. (14)	15	36	M	Myelopathy	VA, OA	MV	Embolization	CO
Reinges et al. (4)	16	58	M	Myelopathy	VA	MV	Surgical disconnection	CO
	17	63	F	Myelopathy	VA	MV	Surgical disconnection	CO
	18	48	M	Myelopathy	VA	MV	Surgical disconnection	CO
Kim et al. (15)	19	36	M	Cerebellar and 4^th^ ventricular hematoma	VA, OA	MV	Embolization	CO
Chng et al. (16)	20	67	M	Myelopathy	APA, OA, VA	Straight sinus	Embolization	Incomplete
Takami et al. (5)	21	69	M	Myelopathy	VA, OA	MV	Surgical disconnection	CO
	22	60	M	Myelopathy	VA	MV	Embolization+ Surgical disconnection	CO
Spiotta et al. (8)	23	49	M	Myelopathy	APA	Cervical radicular vein	Embolization	CO
Liang et al. (7)	24	50	M	Myelopathy	VA, OA	MV	Embolization	CO
	25	61	M	Myelopathy	OA	MV	Embolization	CO
	26	36	M	SAH	VA, OA	MV, Coronal venous plexus	Embolization	CO
	27	55	M	Myelopathy	VA, APA	MV	Embolization	CO
	28	49	F	Myelopathy	VA, APA, OA	MV	Embolization	CO
Gilard et al. (17)	29	59	M	SAH	VA	Superior petrous sinus	Surgical disconnection	CO
Mendes et al. (18)	30	72	M	Occipital hematoma	VA	Marginal sinus, Superior petrosal sinus	Embolization	CO
Pop et al. (19)	31	38	M	Seizure, Myelopathy	APA, OA	ASV, cortical temporal vein	Embolization	CO
Hiramatsu et al. (20)	32	53	M	Myelopathy	APA, OA	PSV	Embolization+ Surgical disconnection	CO
Llacer et al. (21)	33	68	M	Myelopathy	VA, OA	ASV	Embolization	CO
Raheja et al. (22)	34	59	M	Myelopathy	VA	MV	Surgical disconnection	CO
Do et al. (23)	35	57	M	Myelopathy	VA	PSV	Surgical disconnection	CO
Kim et al. (24)	36	48	M	SAH	APA	Suboccipital venous plexus, sigmoid sinus	Embolization	CO
Motebejane et al. (25)	37	45	M	Myelopathy	APA, VA	PSV	Embolization	CO
	38	54	M	Myelopathy	APA, VA	PSV	Embolization	CO
	39	71	M	Myelopathy	APA, VA	ASV, PSV	Embolization	CO
	40	57	M	Myelopathy	APA, VA	ASV	Embolization	CO
	41	47	M	Myelopathy	APA, VA	ASV, PSV	Embolization	CO
	42	67	M	Myelopathy	APA, VA	ASV	Embolization	CO
	43	62	F	Myelopathy	APA, VA	ASV, MV	Embolization	CO
	44	70	M	Myelopathy	APA, VA	ASV	Embolization	CO
	45	49	M	Myelopathy	APA, VA	Pontomesencephalic vein, MV	Embolization	CO
	46	53	M	SAH	APA, VA	Pontomesencephalic vein	Embolization	CO
	47	42	M	SAH	APA, VA	Pontomesencephalic vein	Embolization	CO
	48	51	M	SAH	APA, VA	MV, Jugular bulb	Embolization	CO
Sattur et al. (26)	49	63	F	Myelopathy	VA	ASV	Surgical disconnection	CO
Iampreechakul et al. (10)	50	20	M	Seizure, Medullary hemorrhage	PICA, VA, APA, OA	Petrosal vein, basal vein of Rosenthal, vein of Galen	Embolization additional combined treatment	Incomplete
Chen et al. (9)	51	35	M	Trigeminal neuralgia	APA, OA	Vein of Galen, PSV	Surgical disconnection	CO
Kakizaki, et al. (1)	52	65	M	SAH	APA, VA	MV, anterior pontomesencephalic vein, basal veins of Rosenthal	Embolization + Surgical disconnection	CO
Artemiadis et al. (27)	53	63	M	Myelopathy	APA	MV, ASV	Embolization	No follow up
Gadot, et al. (28)	54	83	M	Myelopathy	VA	MV	Surgical disconnection	CO
Okamoto, et al. (29)	55	50	M	SAH	APA, VA, OA	Straight sinus, superior petrosal sinus	Embolization + surgical disconnection	CO

APA, ascending pharyngeal artery; ASV, anterior spinal vein; CO, complete obliteration; COS, confluence of sinuses; F, female; HASS, Hybrid-Surgical Suite; hOR, hybrid operating room; M, male; MV, medullary vein; OA, Occipital artery; PSV, posterior spinal vein; SAH, subarachnoid hemorrhage; VA, vertebral artery.

### Treatments

Almost all the patients adopted treatments (two patients rejected treatment). Most cases (30/55) were treated with only endovascular embolization, while 18 cases (18/55) were treated with only surgical disconnection, and 5 cases (5/55) with combined therapy (endovascular embolization and microsurgical disconnection) because of incomplete embolization or DAVF reoccurrence.

### Follow-up and outcome

With follow-up duration ranging from 6 months to 2 years, all the treated patients recovered well. All the patients were followed with cerebral DSA. The angiographic outcome of complete obliteration was achieved in all the patients who underwent surgery or combined therapy, and in 30 of 35 patients who received endovascular embolization.

### Illustrative cases

#### Case 12

A 62-year-old male patient presented with a 2-month history of progressive difficulty in walking and bilateral extremity weakness and numbness. He reported no pain but described balance issues and urinary and bowel incontinence episodes. Physical examinations revealed the muscle strength of his distal limbs was grade 4. His Hoffman's and Babinski's signs were both positive. The superficial sensation of his lower body was not intact, with a sensory level at T10.

**Figure 1:**
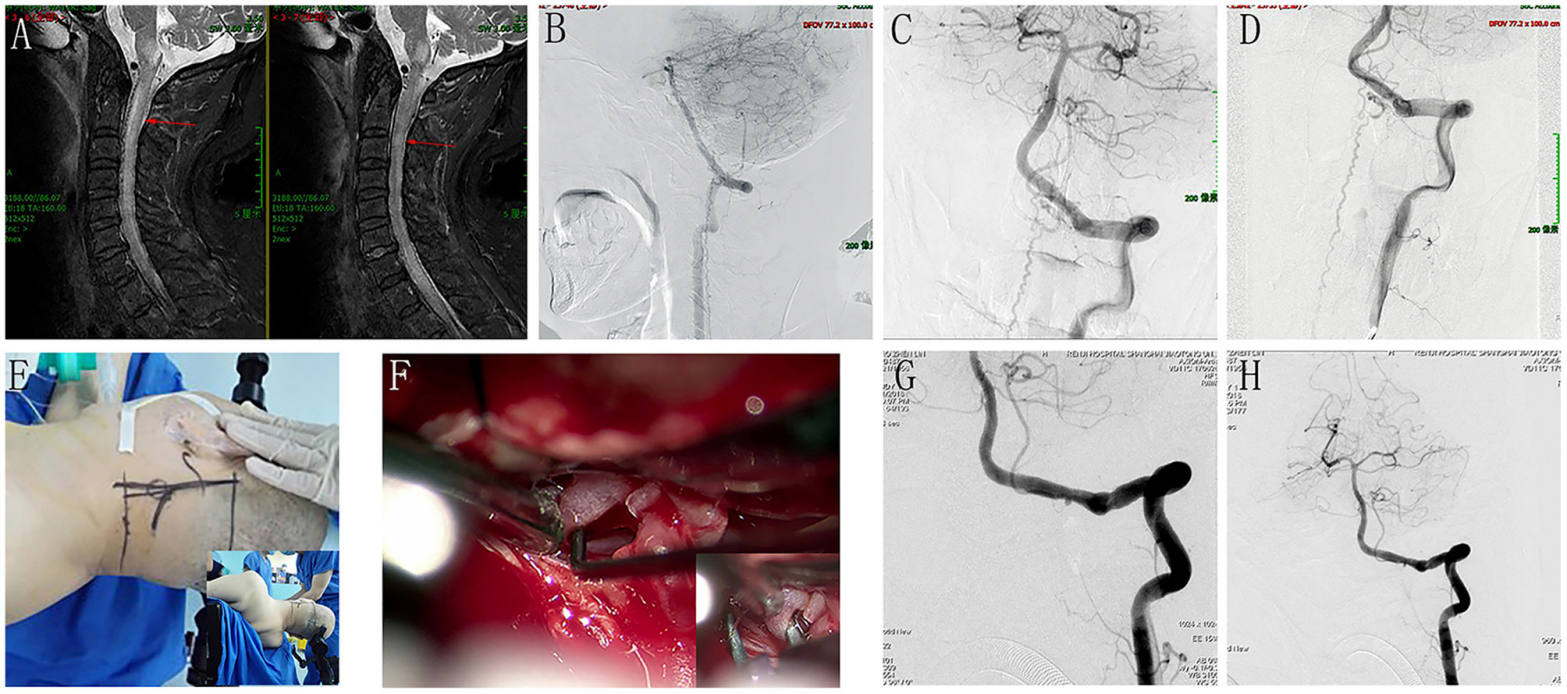
Perioperative neuroimages of Case 12. A cervical spine MRI showed an expanded spinal brain stem and a longitudinal extensive cervical cord enlargement with obvious edema, and vascular flow voids was also demonstrated in front of the cervical cord **(A)**. The cerebral DSA demonstrated a DAVF located at the foramen magnum supplied by the meningeal branch of left VA, and the fistula was draining through the bridge-medullary veins into the dilated tortuous anterior spinal vein **(B)**, lateral view; **(C)**, anteroposterior view; **(D)**, oblique view with magnification. **(E)** showed the incision line and the operation position (park bench position). F demonstrated a large tortuous vein was visible at the level of the foramen magnum, and it originated near the dural penetration of the left vertebral artery and formed a “C” pattern crawling between the vertebral artery and the medulla. **(F)** showed an aneurysm min-clip was temporarily applied to the origin of the fistula to occlude the shunt. Intraoperative DSA revealed the complete disappearance of the DAVF **(G)**. After electrical coagulation and disconnection of the fistula at its origin from the dura, repeat cerebral DSA confirmed the complete obliteration of the fistula **(H)**.

Cervical spine magnetic resonance imaging (MRI) showed an expanded spinal brain stem and a longitudinal extensive cervical cord enlargement with obvious edema. Vascular flow voids were also demonstrated in front of the cervical cord (Figure 1A). The presence of brainstem and cervical cord edema, as well as the vascular flow voids in front of the cervical cord, were strong indicators of a DAVF. The clinicians strongly advised pursuing further angiographic investigations. A complete spine DSA was negative, while the cerebral DSA demonstrated a DAVF located at the foramen magnum supplied by the meningeal branch of the left VA (Figures 1B–D). The fistula was draining through the bridge-medullary veins into the dilated tortuous anterior spinal vein (SPV) (Figures 1B–D; Supplementary material Video 1). After consultation with the multidisciplinary team of the Cerebrovascular Center, a surgical disconnection of the fistula in HASS was considered to be the treatment option. During the operation, the right femoral artery catheterization was first performed. With the patient in the park bench position on his right side, the foramen magnum was osteoclastically enlarged, followed by a partial hemilaminectomy of C1(Figure 1E; Supplementary material Video 2). After opening the dura, a large tortuous vein was visible at the level of the foramen magnum. It originated near the dural penetration of the left vertebral artery and formed a “C” pattern crawling between the vertebral artery and the medulla (Figure 1F). After an aneurysm min-clip was temporarily applied to the origin of the fistula (Figure 1F), intra-operative DSA was performed and revealed the complete disappearance of the DAVF (Figure 1G; Supplementary material Video 3). Thus, electrical coagulation and disconnection of the fistula at its origin from the dura were performed. After the operation, a repeated cerebral DSA confirmed the complete obliteration of the fistula (Figure 1H; Supplementary material Video 4). The postoperative course was uneventful, and his limb's motor and sensory function gradually recovered. Cerebral angiography 6 months later confirmed the disappearance of the DAVF.

#### Case 13

**Figure 2:**
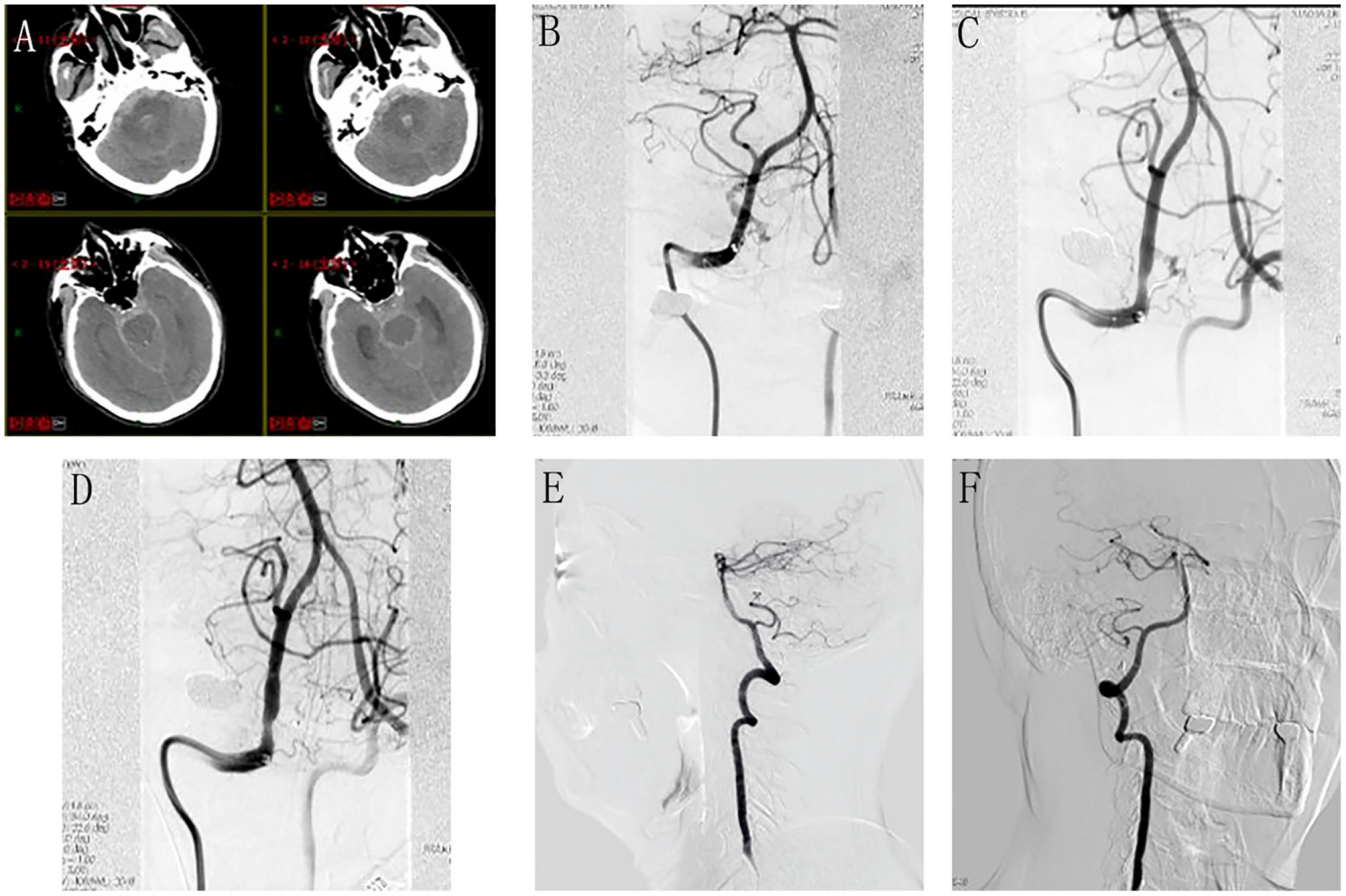
Perioperative neuroimages of Case 13. Emergent cranial CT revealed diffuse SAH and 4th ventricular hematoma **(A)**. Emergent cerebral DSA revealed a DAVF located at the foramen magnum supplied by the meningeal branch of the right VA, draining into the enlarged tortuous medullary vein **(B)**. During the procedure, the microcatheter tip was advanced to reach the optimal position **(C)**; the injection of Onyx was continued until the DAVF was completely obliterated **(D)**. Follow-up angiography at 6 months did not reveal any fistula residual or recurrence **(E)**, lateral view; **(F)**, anteroposterior view.

A 60-year-old male presented with a sudden onset of severe headache and loss of consciousness for 2 h. Emergent cranial CT revealed diffuse SAH and 4th ventricular hematoma (Figure 2A) (Fisher grade IV). The patient was in a deep coma with tracheal intubation (GCS 7, Hunt Hesse IV). Emergent complete cerebral DSA revealed a DAVF located at the foramen magnum supplied by the meningeal branch of the right VA, draining into the enlarged tortuous medullary vein (Figure 2B). As the patient's neurological state was very poor and the only feeding artery was not small, trans-arterial embolization *via* the meningeal branch of the VA in HASS was considered as the treatment option. In addition, microsurgical disconnection of the fistula was regarded as the alternative option, if the endovascular approach failed.

Under general anesthesia, catheterization was performed *via* the transfemoral approach. The Marathon microcatheter (MTI-EV3, Irvine, CA, USA) was dimethylsulfoxide (DMSO)-compatible, and smoothly entered the feeding artery. The microcatheter tip was placed as close as possible to the DAVF nidus to ensure that the liquid embolic agent could penetrate and occlude the lesion. Once the microcatheter tip was advanced to reach the optimal position (Figure 2C), the injection of Onyx-18 (MTI-EV3) was carried out. Whenever any venous migration appeared, the injection was stopped to allow for solidification and subsequently the injection was continued until the DAVF was completely obliterated (Figure 2D). After the embolization, continuous lumbar drainage of bloody cerebrospinal fluid was applied to the patient for 5 days. The patient woke up from a coma 1 week later and recovered well without any neurological deficit 1 month later. Follow-up angiography at 6 months did not reveal any fistula residual or recurrence (Figures 2E, F).

## Discussion

From these 55 cases, foramen magnum DAVFs appeared to be much more common in men (90.9%), with a median age of 52.8 years. Most patients presented with myelopathy or SAH after symptoms appeared, with 30 cases (54.5%) of myelopathy, 21 cases (38.2%) of SAH, and 4 cases (7.3%) of other etiologies (three intracranial hematomas, and one trigeminal neuralgia). The pathophysiological mechanism underlying SAH or myelopathy is presumed to involve venous hypertension, although it is not entirely understood (1, 3, 10). The DAVFs around the foramen magnum could drain into the bridging medullary veins and then to the intracranial cerebral veins or the spinal veins (1). Drainage into the cortical or perimedullary spinal veins increases the pial venous pressure, resulting in aggressive neurologic outcomes including SAH, cerebral hemorrhage, or congestive venous myelopathy (3, 10). Almost all the patients in this study experienced symptoms associated with SAH (38.2%) or myelopathy (54.5%), depending on the pattern of venous drainage.

Anatomically, foramen magnum DAVFs have three important arterial supplies: the neuromeningeal trunk of the APA, the meningeal branches of the VA, and the mastoid branches of the OA (1, 3, 5). As listed in Table 1, among the 55 cases, 21 cases were supplied by only the VA; 3 cases were supplied by only the OA; 3 cases were supplied only by the APA, and the remaining 28 cases were supplied by two or three of these feeding arteries. Therefore, to reduce the incidence of missed or misdiagnosed foramen magnum DAVFs, accurate recognition of six arteries (including the bilateral external carotid arteries, the internal carotid arteries, and the vertebral arteries) and their branches is necessary during the angiography (3).

The dangerous clinical presentations of foramen magnum DAVFs require rapid and aggressive treatment. However, their rarity and complicated angioarchitectures make the treatment difficult and controversial. Generally, microsurgical disconnection of the fistula has been the predominant treatment option for foramen magnum DAVFs before 2010 (3–5). In the past decade, with remarkable advances in endovascular techniques providing highly flexible hydrophilic-coated catheters and new non-adhesive liquid embolic agents, such as Onyx and N-butyl-cyanoacrylate (NBCA) glue, endovascular embolization of the fistula has become an alternative option for treating foramen magnum DAVFs (6–8).

There are abundant anastomoses with surrounding blood vessels in the meningeal branches of VA at the craniocervical junction (1, 3, 5, 30), and embolized materials like Onyx or N-butyl-cyanoacrylate glue (NBCA) can easily migrate or reflux to the basilar and vertebral arteries (1). Therefore, the foramen magnum DAVFs supplied exclusively by the VA are preferably treated with direct microsurgery. As in our current cases, microsurgical disconnection (6/9) was dominantly chosen to treat the foramen magnum DAVFs with VA to be the only feeding artery. On the other hand, in most of the cases supplied by the external carotid artery, the microcatheter was guided sufficiently proximal to the fistula site and the fistula was completely obliterated by using Onyx or NBCA from the external carotid artery. Thus, for DAVFs supplied by the external carotid artery and its branches, endovascular embolization could be preferred (7, 25). During the embolization, the catheter tip should be advanced beyond the hypoglossal or jugular foramina to prevent occlusion of the vasa nervosum.

As shown in Table 1, most cases (30/55) were treated with only endovascular embolization, 18 cases (18/55) with only surgical disconnection, and 5 cases (5/55) with combined therapy (endovascular embolization and microsurgical disconnection) because of incomplete embolization or DAVF reoccurrence. The angiographic outcome of complete obliteration was achieved in all the patients who underwent surgery or combined therapy, and in 30 of 35 patients who received endovascular embolization. Nevertheless, the incomplete obliteration and reoccurrence of the fistula after endovascular embolization could not be underestimated, and some cases even required staged or salvage combined surgeries (1, 10). According to Hiramatsu et al., complication rates of the overall embolization procedure is 15.8% in craniocervical junction DAVF (20). Specifically, cranial nerve palsy may occur after Onyx injection, possibly due to the neurotoxicity of Onyx or dimethyl sulfoxide, or occlusion of the vasa nervorum due to dangerous extracranial-intracranial anastomoses (31–33). Drawing from our experience, for the patients with extracranial-intracranial anastomoses, a non-detachable balloon catheter was inflated in the VA across the C1 branch origin to prevent reflux of the embolized materials into the VA during the embolization.

Notwithstanding alternative treatment options in recent years, microsurgical disconnection of the shunt appears to be a more effective and reliable method of treatment. The surgery can be performed *via* the far lateral suboccipital craniotomy with a C1 laminectomy or hemilaminectomy (3). Microsurgery has also been useful in cases with symptoms caused by the mass effect of the dilated venous pouch and the shunt fistula (1). Chen et al. reported that trigeminal neuralgia due to a foramen magnum DAVF compression was completely relieved after surgical disconnection (9). In addition, although seldom reported, surgery-related complications (intracranial hematoma, intracranial infection, etc.,) cannot be completely avoided.

No treatment is perfect. To achieve complete obliteration of the fistula through minimally invasive procedures, combined therapy might be a better option, especially for the complex foramen magnum DAVFs. However, it often needs staging or additional surgery. Early studies with intraoperative portable DSA (early “hybrid surgery”) showed that intraoperative angiogram altered surgical management, frequently by avoiding additional surgery. Recently, the concept of a Hybrid Angio-Surgical Suite (HASS) has emerged as a solution to the complexity of cerebrovascular surgery and the need for immediate intraoperative feedback (11, 12). It combines the capacity of both the operating room and interventional suite—a standard operating room equipped with a biplane or single-plane angiogram machine. The HASS is becoming a standard facility for many hospitals around the world, and many neurosurgeons and neuro-interventionists use it for the treatment of cerebrovascular diseases (11).

Coincidentally, we treated two cases of foramen magnum DAVFs in HASS with good outcomes. The use of HASS for foramen magnum DAVFs could have the following advantages: 1) the HASS can afford immediate intraoperative feedback (for example, the accurate anatomic location of the fistula, the complete obliteration of the fistula after surgical disconnection); 2) the HASS could immediately provide surgical obliteration when the endovascular approach fails; 3) the HASS with open surgery could supply alternative access for endovascular embolization when the regular trans-arterial and trans-venous approaches fail. In short, HASS provides a less invasive and more feasible alternative for the treatment of difficult foramen magnum DAVFs.

## Conclusion

Foramen magnum DAVFs are rare, with only 55 cases being reported till now. Their angioarchitecture is complicated, with three possible feeder arteries (APA, VA, and OA), draining into the bridging medullary veins and then to the intracranial cerebral veins or the spinal veins, leading to SAH or myelopathy. Their rarity and angio-architectural complexity make their treatment difficult and controversial. Generally, microsurgical disconnection of the fistula appears to be a more effective and reliable method of treatment, while endovascular embolization is a more recent and popular treatment option for selected cases. However, we suggest that all foramen magnum DAVFs should be managed in HASS if available because HASS could provide a less invasive and more feasible alternative treatment strategy.

## Data availability statement

The original contributions presented in the study are included in the article/Supplementary material, further inquiries can be directed to the corresponding author.

## Ethics statement

The studies involving human participants were reviewed and approved by the Ethics Committee of Renji Hospital, School of Medicine, Shanghai Jiaotong University. The patients/participants provided their written informed consent to participate in this study. Written informed consent was obtained from the individual(s) for the publication of any potentially identifiable images or data included in this article.

## Author contributions

LG, ZX, and WG designed the study, analyzed data, and wrote the article. HZ and XZ collected the data. JD and JW assisted in analyzing the data and revised the article. All authors contributed to the article and approved the submitted version.
