# Supplementary material for: Clinical features, angio-architectural phenotypes, and treatment strategy of foramen magnum dural arteriovenous fistulas: a retrospective case series study
Source: Front Neurol. 2023 Apr 18;14:1121075. doi: 10.3389/fneur.2023.1121075 (PMC10151492; doi:10.3389/fneur.2023.1121075)
Supplement: Supplementary file 1 [file Table_1.DOCX]

Table1. Clinical and angioarchitectural Characteristics and treatments of foramen magnum DAVFs

|  | **Case** | **Age** | **Gender** | **Clinical presentation** | **Feeding arteries** | **Venous Drainage** | **Treatment** | **Angiographic Outcome** |
| --- | --- | --- | --- | --- | --- | --- | --- | --- |
| Guo et al. 2010[3] | 1 | 47 | M | SAH | VA | MV | Surgical disconnection | CO |
|  | 2 | 51 | M | SAH | VA | MV | Surgical disconnection | CO |
|  | 3 | 35 | M | SAH | OA, APA | MV, COS | Surgical disconnection | CO |
|  | 4 | 40 | M | SAH | OA | MV, SS, COS | Treatment rejected | No follow up |
| Guo et al. 2012[6] | 5 | 45 | M | SAH | VA | MV, COS | Embolization | CO |
| Present cases | 6 | 30 | M | SAH | VA | MV, COS | Treatment rejected | No follow up |
|  | 7 | 36 | M | SAH | VA | MV | Surgical disconnection | CO |
|  | 8 | 40 | M | SAH | VA | MV | Surgical disconnection | CO |
|  | 9 | 53 | F | SAH | OA | COS | Embolization | CO |
|  | 10 | 52 | M | SAH | VA | COS | Embolization | CO |
|  | 11 | 63 | M | SAH | VA | MV | Surgical disconnection | CO |
|  | 12 | 62 | M | Myelopathy | VA | MV, ASV | Surgical disconnection in HASS | CO |
|  | 13 | 60 | M | SAH | VA | MV | Embolization in HASS | CO |
| Rivierez et al., 1991[13] | 14 | 50 | M | SAH | VA | MV | Surgical disconnection | NR |
| Slaba et al., 2000[14] | 15 | 36 | M | Myelopathy | VA, OA | MV | Embolization | CO |
| Reinges et al., 2001[4] | 16 | 58 | M | Myelopathy | VA | MV | Surgical disconnection | CO |
|  | 17 | 63 | F | Myelopathy | VA | MV | Surgical disconnection | CO |
|  | 18 | 48 | M | Myelopathy | VA | MV | Surgical disconnection | CO |
| Kim et al., 2003[15] | 19 | 36 | M | Cerebellar and 4^th^ ventricular hematoma | VA, OA | MV | Embolization | CO |
| Chng et al., 2004[16] | 20 | 67 | M | Myelopathy | APA, OA, VA | Straight sinus | Embolization | Incomplete |
| Takami et al., 2005[5] | 21 | 69 | M | Myelopathy | VA, OA | MV | Surgical disconnection | CO |
|  | 22 | 60 | M | Myelopathy | VA | MV | Embolization+ Surgical disconnection | CO |
| Spiotta et al., 2011[8] | 23 | 49 | M | Myelopathy | APA | Cervical radicular vein | Embolization | CO |
| Liang et al., 2013[7] | 24 | 50 | M | Myelopathy | VA, OA | MV | Embolization | CO |
|  | 25 | 61 | M | Myelopathy | OA | MV | Embolization | CO |
|  | 26 | 36 | M | SAH | VA, OA | MV, Coronal venous plexus | Embolization | CO |
|  | 27 | 55 | M | Myelopathy | VA, APA | MV | Embolization | CO |
|  | 28 | 49 | F | Myelopathy | VA, APA, OA | MV | Embolization | CO |
| Gilard et al., 2013[17] | 29 | 59 | M | SAH | VA | Superior petrous sinus | Surgical disconnection | CO |
| Mendes et al., 2015[18] | 30 | 72 | M | Occipital hematoma | VA | Marginal sinus, Superior petrosal sinus | Embolization | CO |
| Pop et al., 2015[19] | 31 | 38 | M | Seizure, Myelopathy | APA, OA | ASV, cortical temporal vein | Embolization | CO |
| Hiramatsu et al., 2015[20] | 32 | 53 | M | Myelopathy | APA, OA | PSV | Embolization+ Surgical disconnection | CO |
| Llacer et al., 2016[21] | 33 | 68 | M | Myelopathy | VA, OA | ASV | Embolization | CO |
| Raheja et al., 2017[22] | 34 | 59 | M | Myelopathy | VA | MV | Surgical disconnection | CO |
| Do et al., 2017[23] | 35 | 57 | M | Myelopathy | VA | PSV | Surgical disconnection | CO |
| Kim et al., 2018[24] | 36 | 48 | M | SAH | APA | Suboccipital venous plexus, sigmoid sinus | Embolization | CO |
| Motebejane et al., 2018[25] | 37 | 45 | M | Myelopathy | APA, VA | PSV | Embolization | CO |
|  | 38 | 54 | M | Myelopathy | APA, VA | PSV | Embolization | CO |
|  | 39 | 71 | M | Myelopathy | APA, VA | ASV, PSV | Embolization | CO |
|  | 40 | 57 | M | Myelopathy | APA, VA | ASV | Embolization | CO |
|  | 41 | 47 | M | Myelopathy | APA, VA | ASV, PSV | Embolization | CO |
|  | 42 | 67 | M | Myelopathy | APA, VA | ASV | Embolization | CO |
|  | 43 | 62 | F | Myelopathy | APA, VA | ASV, MV | Embolization | CO |
|  | 44 | 70 | M | Myelopathy | APA, VA | ASV | Embolization | CO |
|  | 45 | 49 | M | Myelopathy | APA, VA | Pontomesencephalic vein, MV | Embolization | CO |
|  | 46 | 53 | M | SAH | APA, VA | Pontomesencephalic vein | Embolization | CO |
|  | 47 | 42 | M | SAH | APA, VA | Pontomesencephalic vein | Embolization | CO |
|  | 48 | 51 | M | SAH | APA, VA | MV, Jugular bulb | Embolization | CO |
| Sattur et al., 2019[26] | 49 | 63 | F | Myelopathy | VA | ASV | Surgical disconnection | CO |
| Iampreechakul et al., 2019 [10] | 50 | 20 | M | Seizure, Medullary hemorrhage | PICA, VA, APA, OA | Petrosal vein, basal vein of Rosenthal, vein of Galen | Embolization additional combined treatment | Incomplete |
| Chen et al., 2010[9] | 51 | 35 | M | Trigeminal neuralgia | APA, OA | Vein of Galen, PSV | Surgical disconnection | CO |
| Kakizaki, et al., 2020[1] | 52 | 65 | M | SAH | APA, VA | MV, anterior pontomesencephalic vein, basal veins of Rosenthal | Embolization + Surgical disconnection | CO |
| Artemiadis et al., 2020[27] | 53 | 63 | M | Myelopathy | APA | MV, ASV | Embolization | No follow up |
| Gadot, et al., 2021[28] | 54 | 83 | M | Myelopathy | VA | MV | Surgical disconnection | CO |
| Okamoto, et al., 2021[29] | 55 | 50 | M | SAH | APA, VA, OA | Straight sinus, superior petrosal sinus | Embolization + surgical disconnection | CO |

**APA, ascending pharyngeal artery; ASV, anterior spinal vein; CO, complete obliteration; COS, confluence of sinuses; F, female; HASS, Hybrid-Surgical Suite; hOR, hybrid operating room; M, male; MV, medullary vein; OA, Occipital artery; PSV, posterior spinal vein; SAH, subarachnoid hemorrhage; VA, vertebral artery.**
